# Imposed Upon

**Published:** 1876-04

**Authors:** 


ALWAYS read our advertisements, and present their claims to your home druggist.

*Imposed Upon.*—It becomes our duty to inform our readers that the advertisement in our last headed "*Birds*," by one F. E. G. Lindsey, Abingdon, Va., is an imposition, as we learn from a reliable party in that section. We inserted without our usual caution, not suspecting that in the matter of *parrots, fowls, guinea pigs,* etc., that any one would attempt a fraud. Our information, however, is reliable that such is the case, and we therefore warn our readers to steer clear of the aforesaid Lindsey.

*American Medical Association.*—The twenty-seventh annual session of the above body will be held in Philadelphia June 6th, 1876. In the same city the *International Medical Congress* will be formally opened on the 4th day of September, at noon, in the University of Pennsylvania. In time we will give further particulars. *Georgia Medical Association* will assemble in Augusta on the 19th inst. The railroads will pass members at half-rates.

